# Supplementary figures and images for: Prebiotic gas flow environment enables isothermal nucleic acid replication
Source: eLife. 2025 Jul 9;13:RP100152. doi: 10.7554/eLife.100152 (PMC12240584; doi:10.7554/eLife.100152)

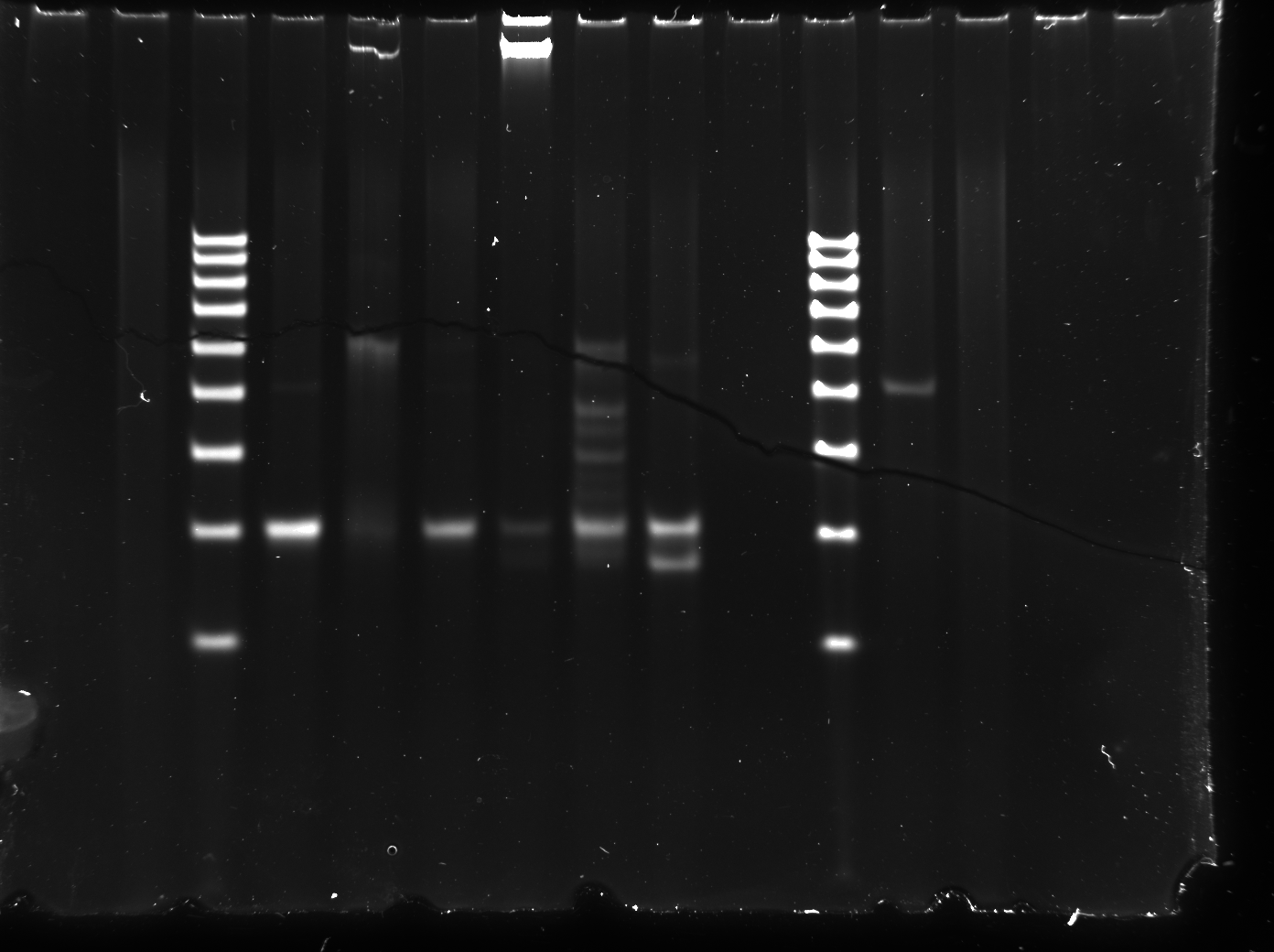

Supplement: Figure 4—source data 1. [file elife-100152-fig4-data1.zip › Figure 4-source data 1/Figure 4 b)- Lane 1-4.tif]

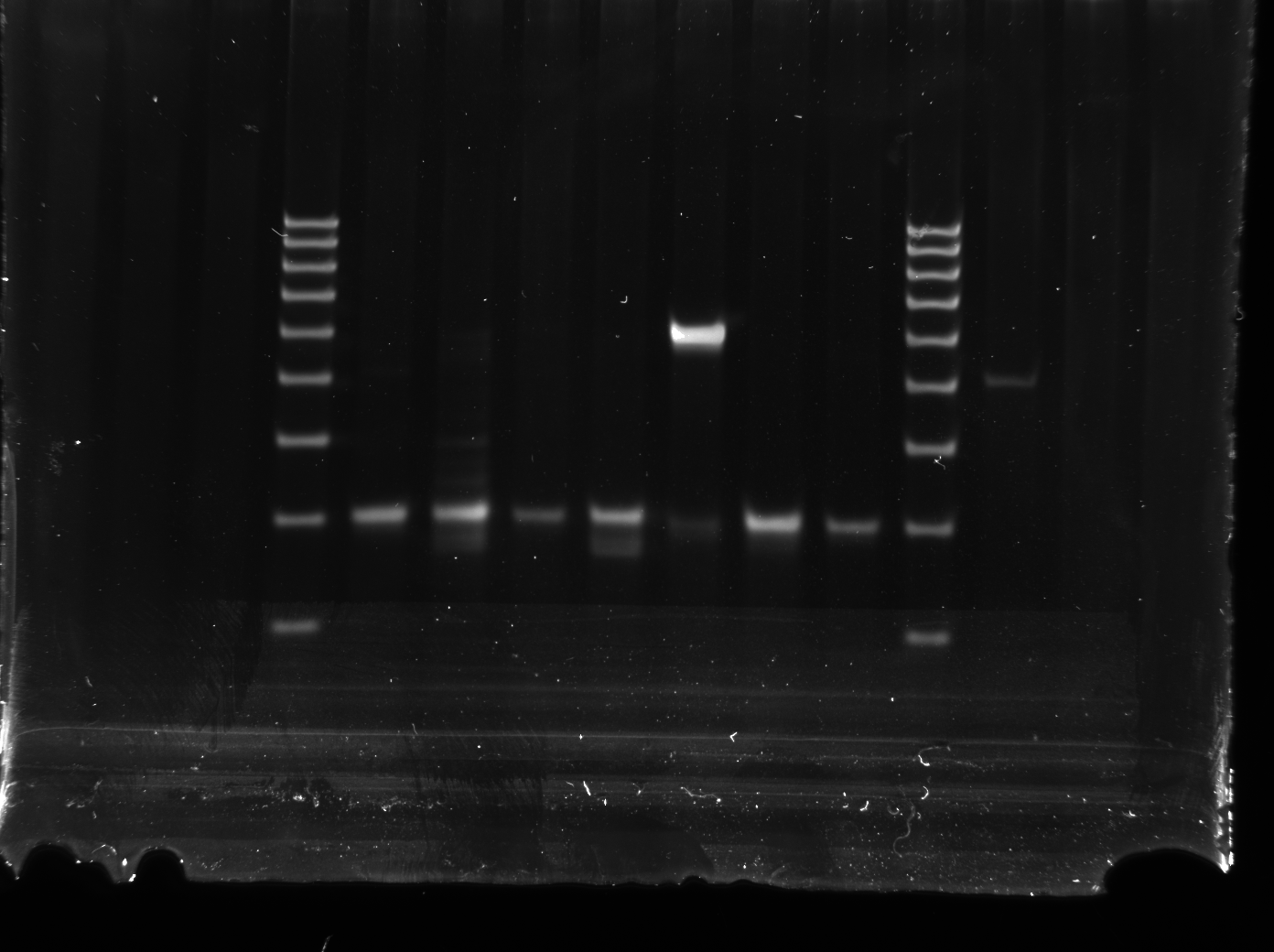

Supplement: Figure 4—source data 1. [file elife-100152-fig4-data1.zip › Figure 4-source data 1/Figure 4 b)- Lane 5-6.tif]

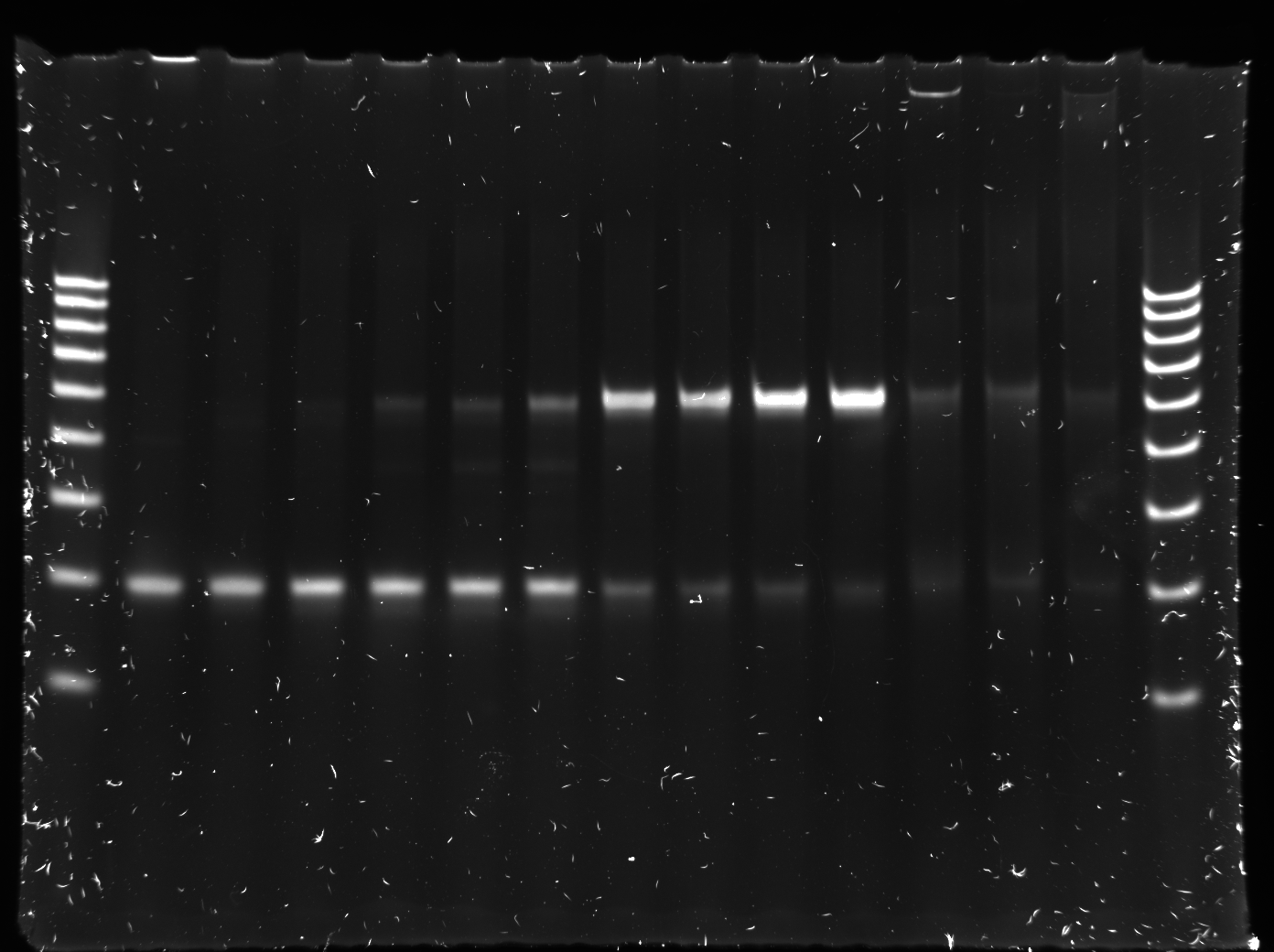

Supplement: Figure 4—source data 1. [file elife-100152-fig4-data1.zip › Figure 4-source data 1/Figure 4 b)- Lane 7.tif]

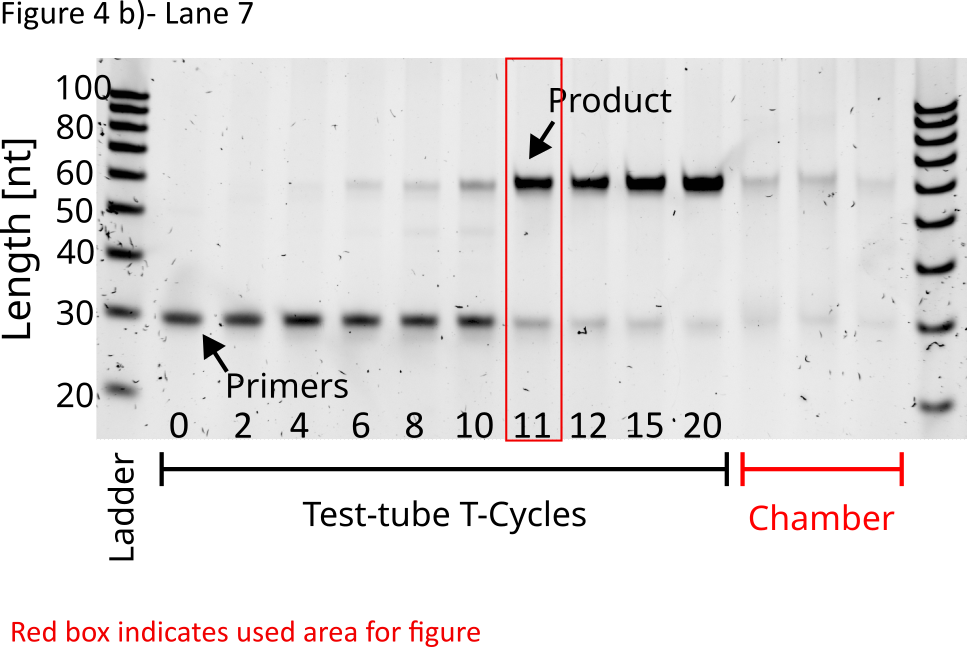

Supplement: Figure 4—source data 2. [file elife-100152-fig4-data2.zip › Figure 4-source data 2/Figure 4 b)- Lane 7.png]

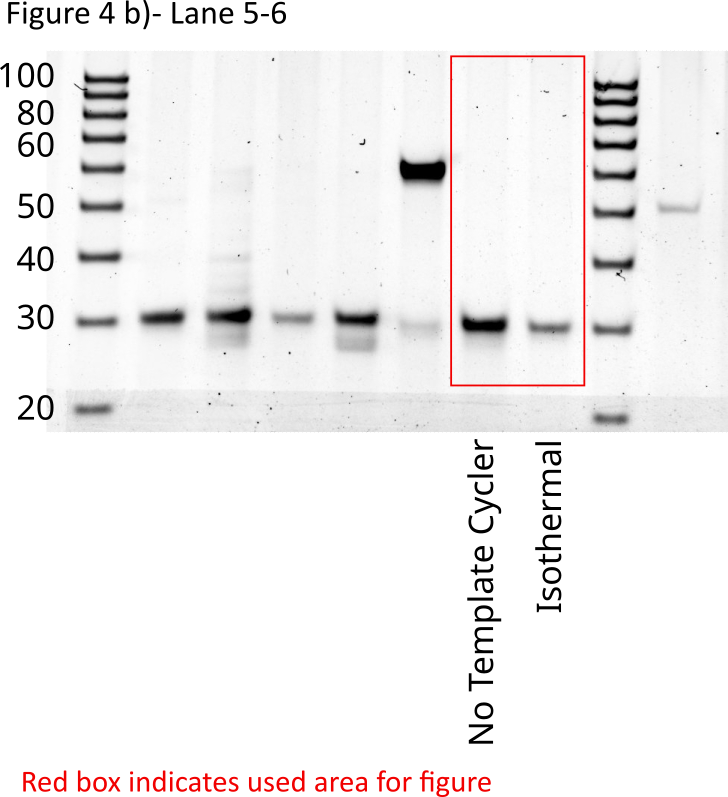

Supplement: Figure 4—source data 2. [file elife-100152-fig4-data2.zip › Figure 4-source data 2/Figure 4 b)- Lanes 5-6.png]

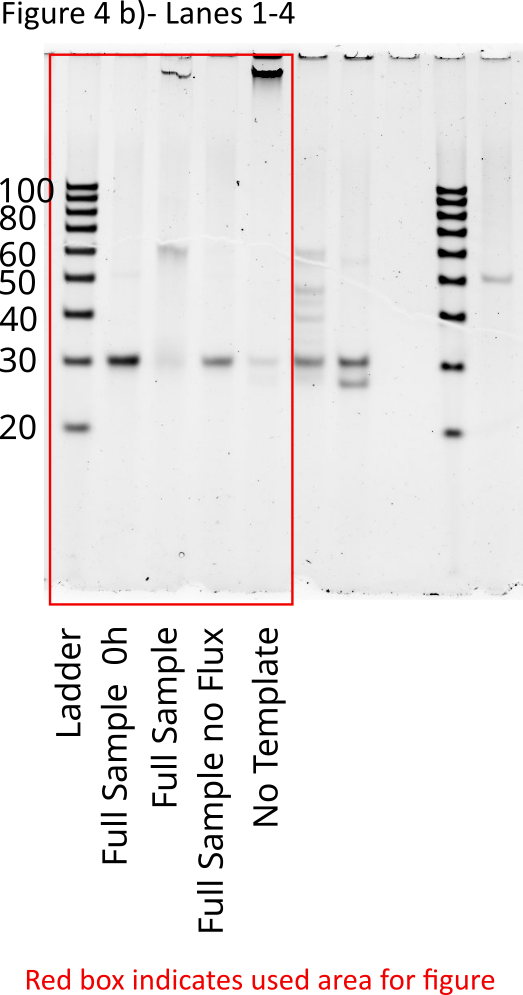

Supplement: Figure 4—source data 2. [file elife-100152-fig4-data2.zip › Figure 4-source data 2/Figure 4 b)- Lnes 1-4.png]

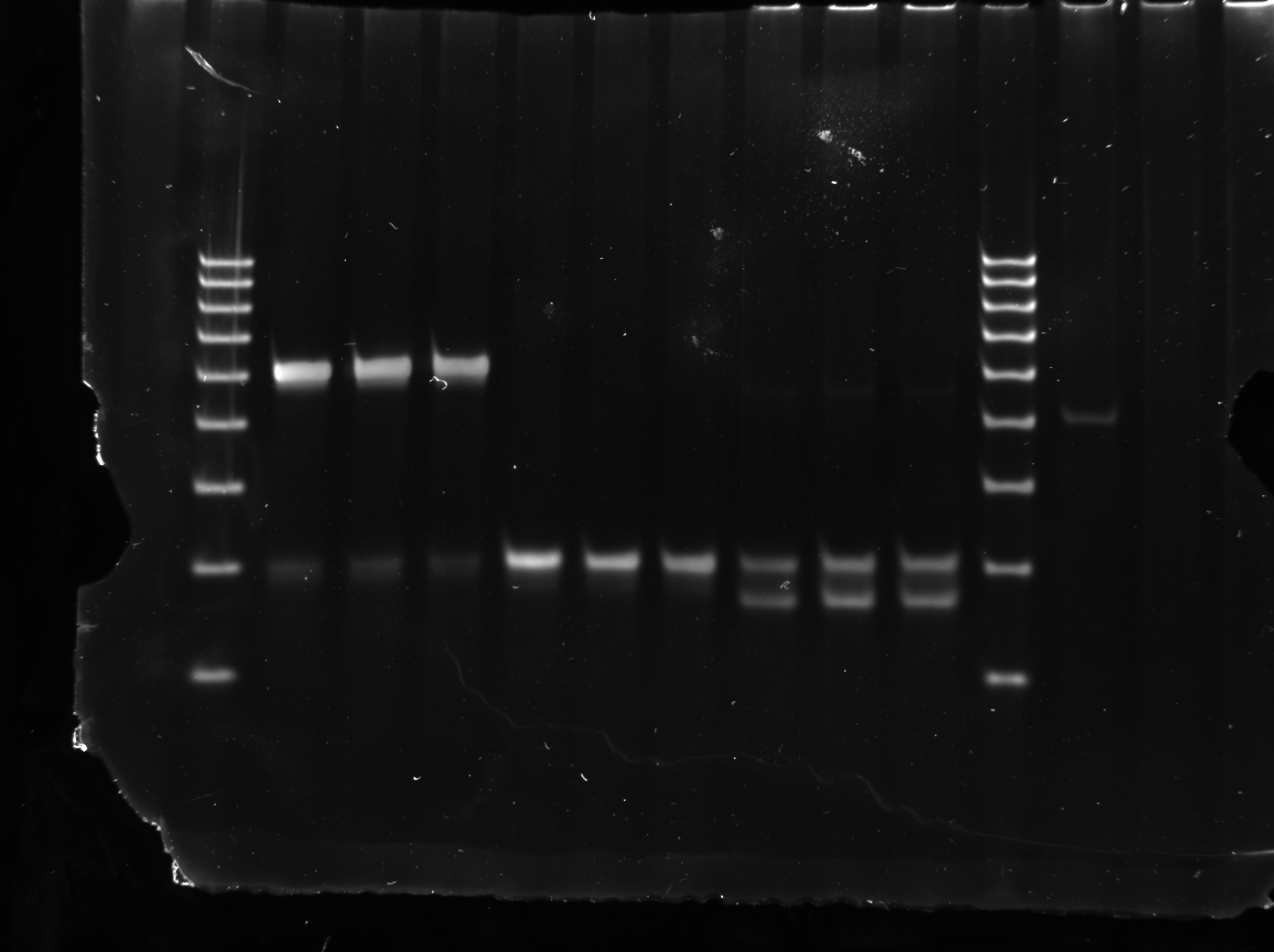

Supplement: Figure 4—figure supplement 2—source data 1. [file elife-100152-fig4-figsupp2-data1.zip › Figure 4- figure supplement 2-source data 1/Figure 4- figure supplement 2 c) Left Panel.tif]

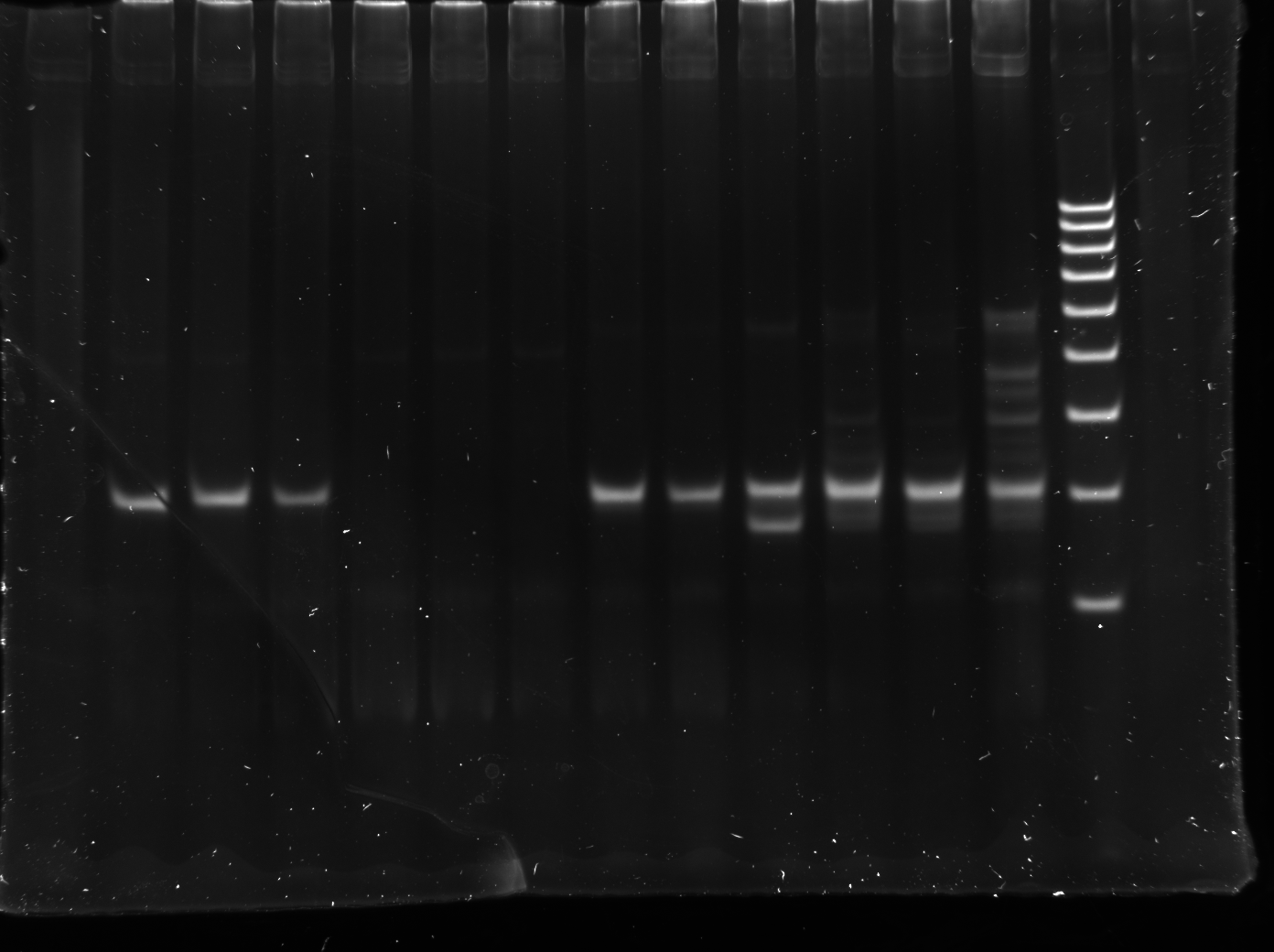

Supplement: Figure 4—figure supplement 2—source data 1. [file elife-100152-fig4-figsupp2-data1.zip › Figure 4- figure supplement 2-source data 1/Figure 4- figure supplement 2 c) Right Panel (2).tif]

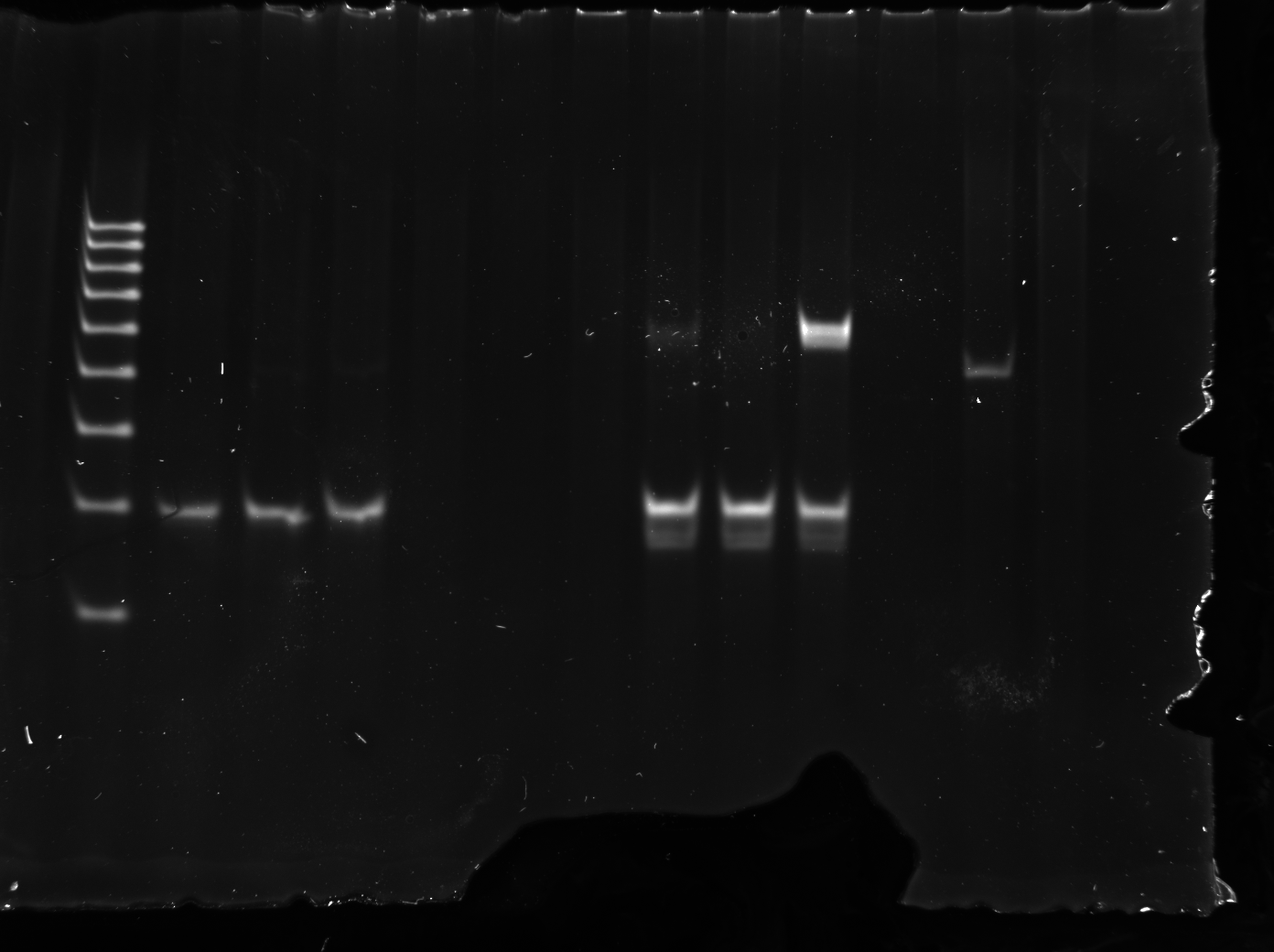

Supplement: Figure 4—figure supplement 2—source data 1. [file elife-100152-fig4-figsupp2-data1.zip › Figure 4- figure supplement 2-source data 1/Figure 4- figure supplement 2c) Right Panel (1).tif]

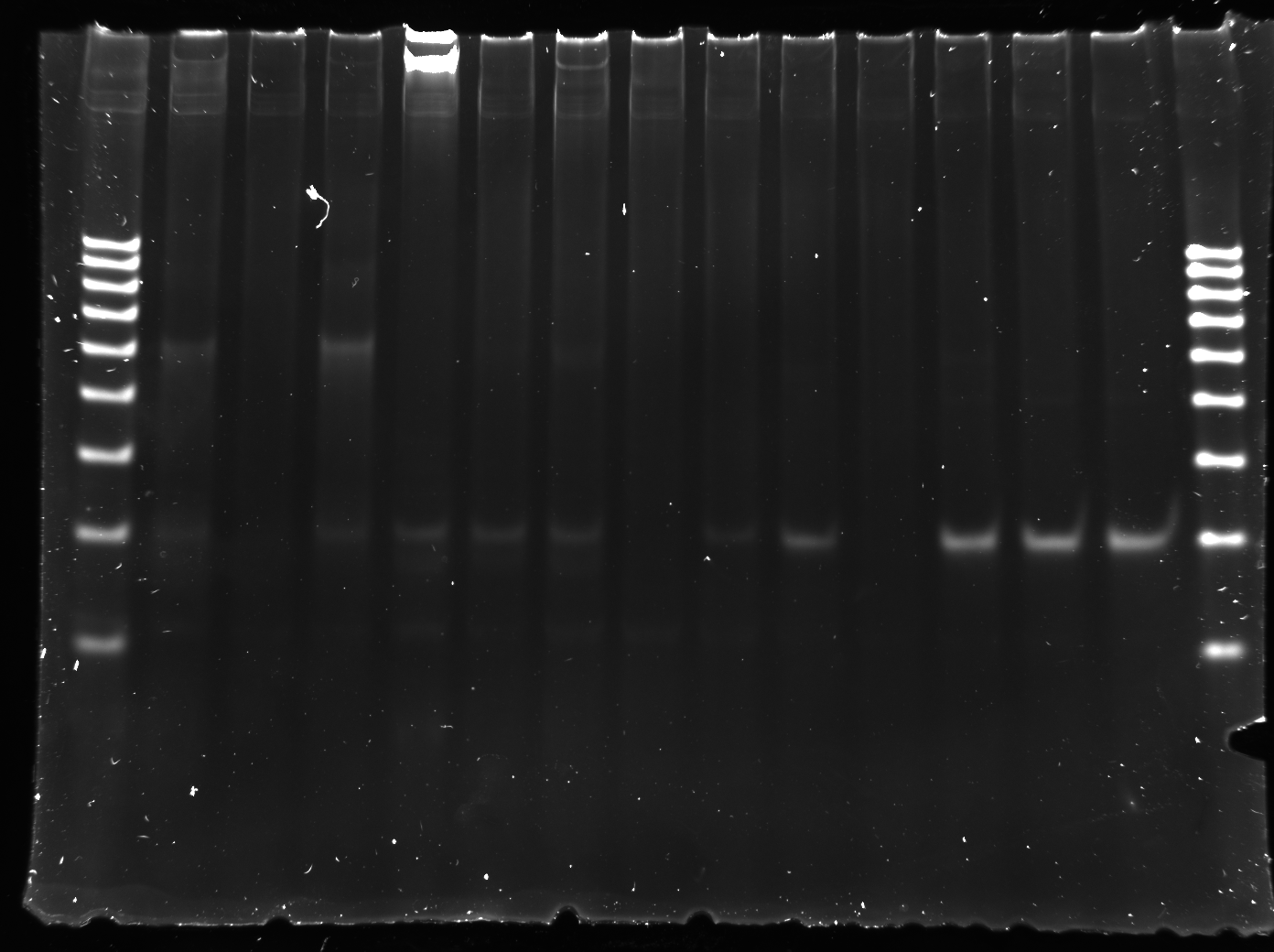

Supplement: Figure 4—figure supplement 2—source data 1. [file elife-100152-fig4-figsupp2-data1.zip › Figure 4- figure supplement 2-source data 1/Figure 4- figure supplement 2d).tif]

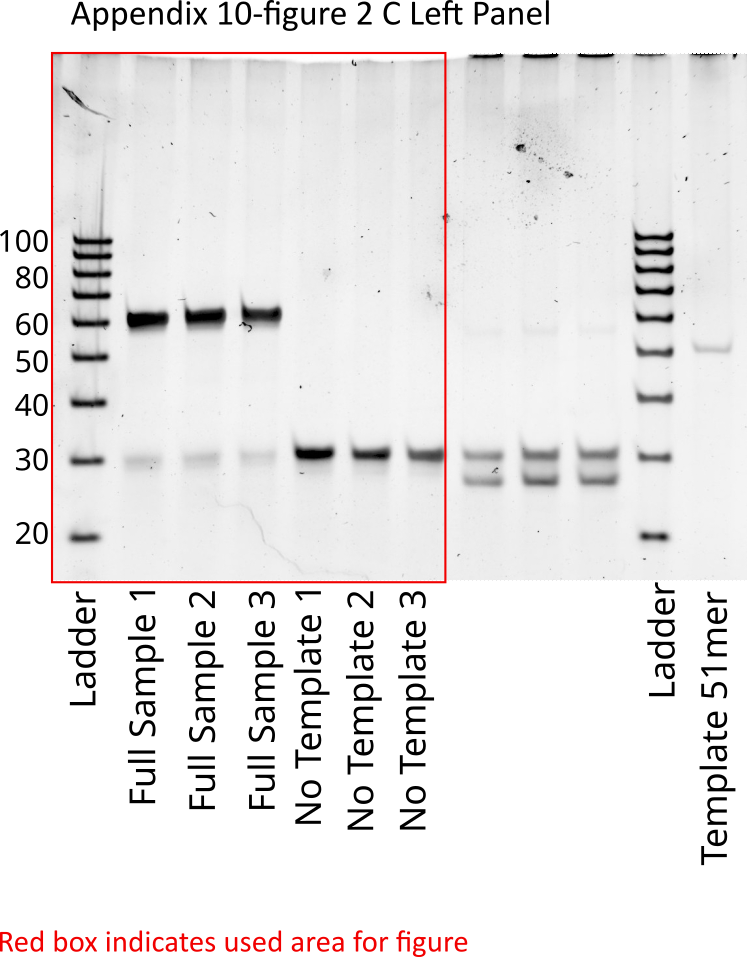

Supplement: Figure 4—figure supplement 2—source data 2. [file elife-100152-fig4-figsupp2-data2.zip › Figure 4- figure supplement 2-source data 2/Figure 4- figure supplement 2 c) Left Panel.png]

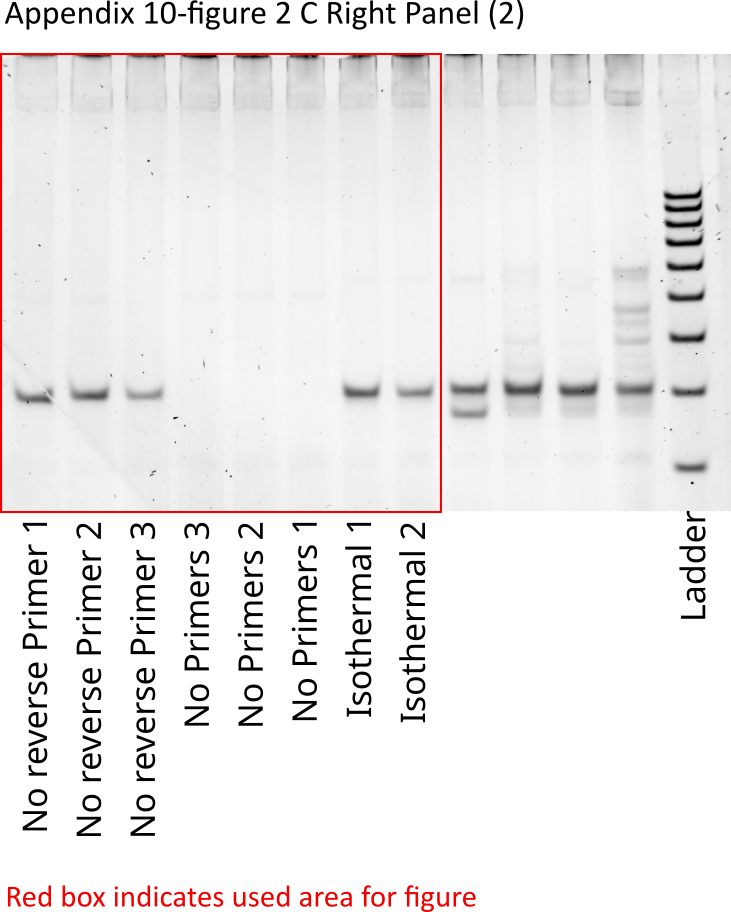

Supplement: Figure 4—figure supplement 2—source data 2. [file elife-100152-fig4-figsupp2-data2.zip › Figure 4- figure supplement 2-source data 2/Figure 4- figure supplement 2 c) Right Panel (2).png]

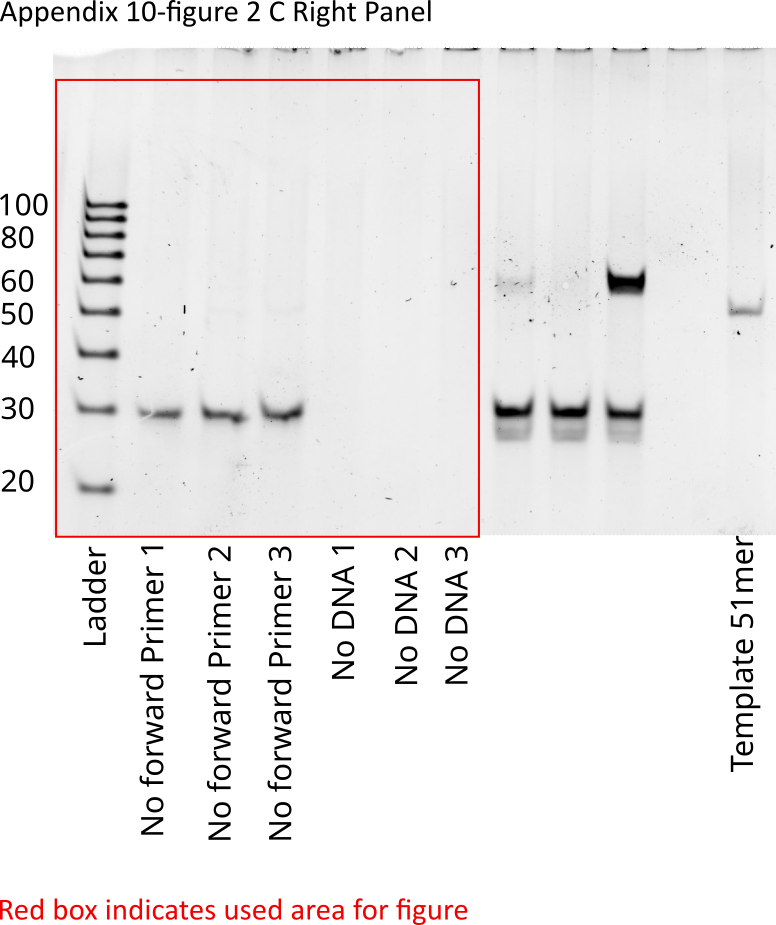

Supplement: Figure 4—figure supplement 2—source data 2. [file elife-100152-fig4-figsupp2-data2.zip › Figure 4- figure supplement 2-source data 2/Figure 4- figure supplement 2 c) Right Panel.png]

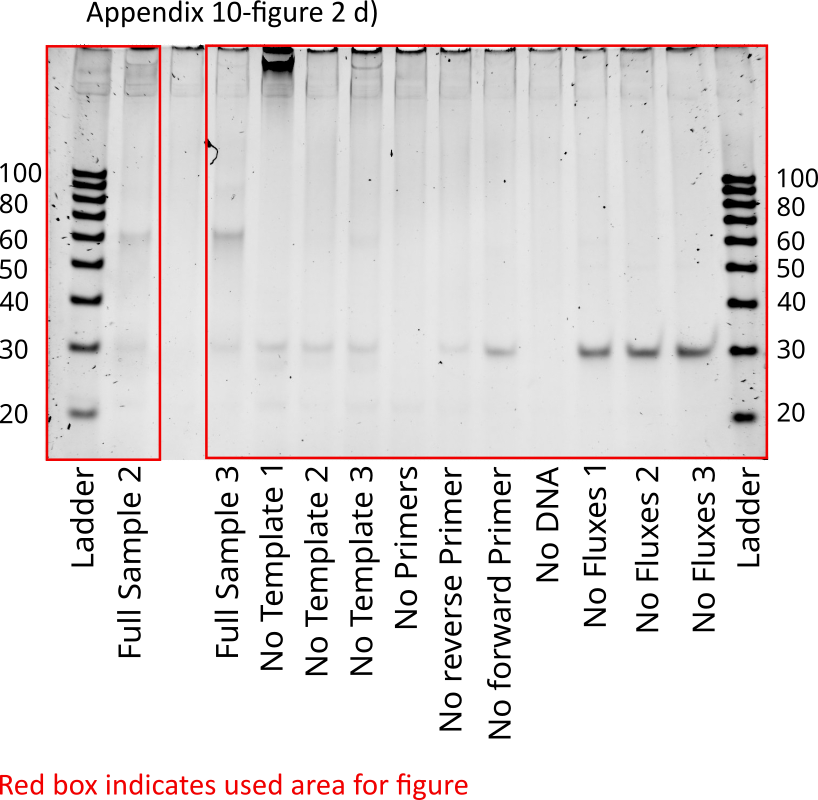

Supplement: Figure 4—figure supplement 2—source data 2. [file elife-100152-fig4-figsupp2-data2.zip › Figure 4- figure supplement 2-source data 2/Figure 4- figure supplement 2d).png]

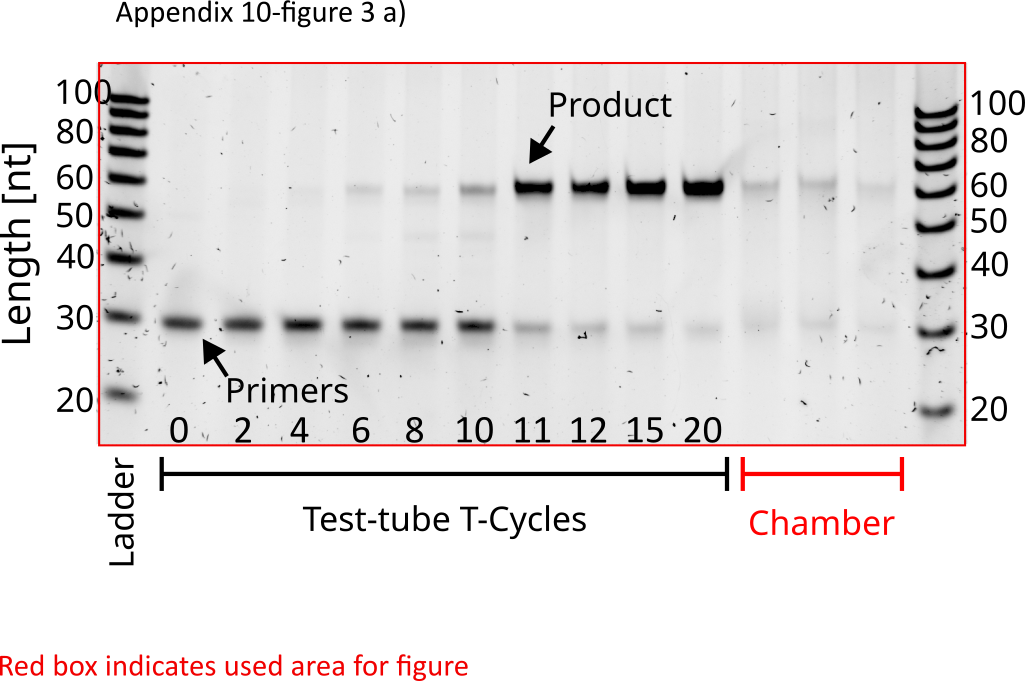

Supplement: Figure 4—figure supplement 3—source data 2. [file elife-100152-fig4-figsupp3-data2.zip › Figure 4- figure supplement 3-source data 2/Figure 4- figure supplement 3 a).png]
